# Genotype imputation for soybean nested association mapping population to improve precision of QTL detection

**DOI:** 10.1007/s00122-022-04070-7

**Published:** 2022-03-11

**Authors:** Linfeng Chen, Shouping Yang, Susan Araya, Charles Quigley, Earl Taliercio, Rouf Mian, James E. Specht, Brian W. Diers, Qijian Song

**Affiliations:** 1 Soybean Genomics and Improvement Laboratory, United States Department of Agriculture, Agricultural Research Service, Beltsville Agricultural Research Center, Beltsville, MD 20705 USA; 2 National Center for Soybean Improvement, Key Laboratory of Biology and Genetic Improvement of Soybean, Ministry of Agriculture, State Key Laboratory of Crop Genetics and Germplasm Enhancement, Jiangsu Collaborative Innovation Center for Modern Crop Production, College of Agriculture, Soybean Research Institute, Nanjing Agricultural University, Nanjing, 210095 China; 3 Soybean and Nitrogen Fixation Research, USDA-ARS, Raleigh, NC 27607 USA; 4 Department of Agronomy and Horticulture, University of Nebraska, Lincoln, NE 68583 USA; 5 Department of Crop Sciences, National Soybean Research Center, University of Illinois, 1101 West Peabody Drive, Urbana, IL 61801 USA

## Abstract

**Key message:**

**Software for high imputation accuracy in soybean was identified. Imputed dataset could significantly reduce the interval of genomic regions controlling traits, thus greatly improve the efficiency of candidate gene identification.**

**Abstract:**

Genotype imputation is a strategy to increase marker density of existing datasets without additional genotyping. We compared imputation performance of software BEAGLE 5.0, IMPUTE 5 and AlphaPlantImpute and tested software parameters that may help to improve imputation accuracy in soybean populations. Several factors including marker density, extent of linkage disequilibrium (LD), minor allele frequency (MAF), etc., were examined for their effects on imputation accuracy across different software. Our results showed that AlphaPlantImpute had a higher imputation accuracy than BEAGLE 5.0 or IMPUTE 5 tested in each soybean family, especially if the study progeny were genotyped with an extremely low number of markers. LD extent, MAF and reference panel size were positively correlated with imputation accuracy, a minimum number of 50 markers per chromosome and MAF of SNPs > 0.2 in soybean line were required to avoid a significant loss of imputation accuracy. Using the software, we imputed 5176 soybean lines in the soybean nested mapping population (NAM) with high-density markers of the 40 parents. The dataset containing 423,419 markers for 5176 lines and 40 parents was deposited at the Soybase. The imputed NAM dataset was further examined for the improvement of mapping quantitative trait loci (QTL) controlling soybean seed protein content. Most of the QTL identified were at identical or at similar position based on initial and imputed datasets; however, QTL intervals were greatly narrowed. The resulting genotypic dataset of NAM population will facilitate QTL mapping of traits and downstream applications. The information will also help to improve genotyping imputation accuracy in self-pollinated crops.

**Supplementary Information:**

The online version contains supplementary material available at 10.1007/s00122-022-04070-7.

## Introduction

In modern breeding programs, germplasm is frequently required to be genotyped with mega- or giga-sized sets of single nucleotide polymorphism (SNP) markers. Although the cost of genotyping-by-sequencing (GBS) and BeadChips-based SNP assays significantly reduced over past years, it is still costly for either large scale genomic studies or most molecular breeding programs in crop plants, including soybean. Genotype imputation is a method to infer missing genotypes of markers residing in intervals flanked by fully informative markers in a study dataset based on the haplotype information from a reference dataset. It enables researchers to obtain a dataset with dense genotypes from skim sequencing or low marker density array datasets (Browning et al. 2018). Imputed genotypes can be used to improve statistical power and resolution of gene mapping in genome-wide association studies (GWAS) or genetic linkage mapping and to integrate multi-studies in meta-analysis (Marchini and Howie 2010; MacArthur et al. 2017; Mahajan et al. 2018).

Genotype imputation leverages the shared haplotype information between a study panel and a reference panel to estimate variants in study individuals that are not directly genotyped (Yun et al. 2009). Imputation algorithms can be divided into two main categories: population-based imputation and pedigree-based imputation. Population-based imputation relies on linkage disequilibrium (LD) to infer missing genotypes of unrelated individuals in the study panel (Stephens and Scheet 2005). The shared haplotype stretches among these unrelated individuals tend to be very short because their common ancestors are distant. As the number of haplotypes in a reference panel increases, a given haplotype in each study panel has fewer longer stretches of matching haplotype within the reference haplotype blocks, thereby improving the imputation accuracy (Browning and Browning 2016). Many imputation algorithms and software packages such as MINIMAC4, BEAGLE 5.0 and IMPUTE 5 have been developed to increase computational efficiency without loss of imputation accuracy (Das et al. 2016; Browning et al. 2018; Rubinacci et al. 2019). Pedigree-based imputation relies on regions of identical-by-descent (IBD) between individuals with pedigree information, missing genotypes are inferred by comparing incompletely genotyped individual haplotypes with the haplotypes that are IBD in the family (Yun et al. 2009). The accuracy of pedigree-based imputation is theoretically higher than population-based imputation because family members share longer haplotype stretches (Hickey et al. 2015; Antolín et al. 2017) due to limited recombination events (Song et al. 2017). Bi-parental populations are typical family populations and are commonly available in most public plant breeding programs. It is cost-effective to genotype just the two parents at high marker density and use their genotypes as references to impute genotypes of low-marker density assayed progeny because parental homozygosity confers de-facto haplotype phasing (Hickey et al. 2015). Several imputation tools, e.g., BEAGLE and IMPUTE, have undergone successive versions of optimization. BEAGLE 5.0 uses haplotype frequency model described by Li and Stephens (2003) with a highly parsimonious algorithm to construct a small subset of reference haplotype from a full reference panel for imputation, which enables to the use of large reference panels with a significant reduction in computational cost in imputation (Browning et al. 2018). Although it is a more computationally intensive imputation method, the current version of IMPUTE 5 is now greatly improved in speed, accuracy and memory efficiency by using new reference panel file format and haplotype-selecting strategy based on the Positional Burrows Wheeler Transform (PBWT) (Rubinacci et al. 2019). BEAGLE 5.0 and IMPUTE 5 were originally developed for use in human populations which have high genetic diversity and a limited number of similar haplotype clusters (Pook et al. 2020); thus, its default parameters were intrinsically developed to work for that type of genetic structure. In contrast, soybean is an inbred crop with relatively low genetic diversity and a long stretch of related haplotypes, especially in the bi-parental derived populations. Although both software models have been widely used in animal and plant genetics, parameters affecting the size of haplotype cluster in the study panel of inbred plant like soybean need to be optimized. AlphaPlantImpute is specifically designed for imputing diploid bi-parental populations with ungenotyped markers and explicitly leverages the characteristics of plant breeding programs, which usually involve highly homozygous parents, high-density marker genotyping of parents, low-density marker genotyping of progeny and large stretches of parental haplotype in progeny (Gonen et al. 2018). Other tools specifically designed to integrate GBS data from bi-parental populations in plants, including Tassel-FSFHap (Swarts et al. 2014), LB-impute (Fragoso et al. 2016), and NOISYmputer (Lorieux et al. 2019), are also available.

Linkage mapping is a powerful tool to detect associations between genetic and phenotypic variation in recombinant inbred line (RIL) population, but the resolution of quantitative trait loci (QTL) map position is usually poor due to the limited opportunity for recombination to occur during the selfing generations from F_1_- to F_*n*_-derived RIL generations and the potential lack of diversity between mated parental pair. GWAS can partially resolve this problem because it is conducted using a large number of germplasm accessions of unknown pedigree, and thus provides a higher mapping resolution by exploiting historical recombination events that were the presumed causal basis giving rise to diversity among population members. However, GWAS exhibits lower power than QTL mapping (Kingsmore et al. 2008) and results can be confounded by population structure (Yu et al. 2006). Nested association mapping (NAM) combines the strengths of both linkage mapping and association mapping to increase statistical power, improve mapping resolution while decreasing confounding population structure (Yu et al. 2008). NAM populations are designed by crossing multiple diverse founders to a common parent followed by generations of selfing in each family. Although high-density genotyping for thousands of RILs can greatly improve the accuracy and precision of QTL discovery, genotyping of the large population is costly. A more cost-effective strategy is to genotype the NAM parents via high-throughput deep sequencing or high-density array analysis while genotyping the segregating progeny with low-density set of markers, followed by imputing the ungenotyped markers in the progeny using the parental genotypic dataset as the reference. The imputation can be performed in every single family because the parents are sufficient to define the genetic structure of the segregation progeny (Yu et al. 2008). For a rice NAM GBS dataset, missing parental genotypes were first imputed based on sequenced offspring, then the imputed parental dataset was filtered and used as reference to impute the offspring in each of the 10 families with LB-impute (Fragoso et al. 2017). In sorghum NAM population, Full-Sib Family Haplotype Imputation (FSFHap) method was used to impute missing genotypes in the NAM RILs in full-sib families, while missing genotypes in diverse accessions were imputed with BEAGLE 4 (Bouchet et al. 2017).

A soybean NAM population, consisting of 5600 RILs from 40 families has been genetically characterized (Song et al. 2017) and used to dissect complex traits including disease resistance of *Phytophthora sojae* (Scott et al. 2019), drought tolerance (Buezo et al. 2019), yield and other important agronomic traits (Diers et al. 2018). In this NAM population, 41 parents were genotyped by GBS and SoySNP50K BeadChip assay (Song et al. 2013, 2015), while 5600 RILs were genotyped by SoyNAM6K BeadChip with 4312 SNPs. Although the dataset of the RILs and parents genotyped with 4312 SNPs is available at the Soybase (https://soybase.org/SoyNAM/), a high-quality imputed dataset containing high density of SNPs for the NAM RILs has not been created. Thus, the objectives of this study were to evaluate imputation performance of the three commonly used imputation software, BEAGLE, IMPUTE and AlphaPlantImpute in soybean populations considering a number of factors including the number of markers in the study panel, extent of LD, minor allele frequency (MAF) of markers and genetic map distance vs. physical distance, to generate soybean NAM RIL imputed genotype dataset with optimized software parameters for public utilization and to demonstrate QTL mapping improvement based on the imputed RILs dataset vs. original dataset in linkage mapping analysis.

## Materials and method

### Genotype datasets

The genotypic dataset analyzed in this study was obtained from a NAM population, which was developed by crossing 40 diverse soybean genotypes to the common cultivar followed by the development of 140 F_5_-derived RILs in each family (Diers et al. 2018). The 41 NAM parents were deeply sequenced using the Illumina HiSeq 2000 (Song et al. 2017) and assayed with the SoySNP50K BeadChip (Song et al. 2013), and the 5600 RILs together with their parents were genotyped with the SoyNAM6K BeadChip containing 6 K SNPs which were selected from the SNPs in sequencing analysis among 41 parents based on their polymorphism, genomic position, uniqueness of their flanking sequence and other criteria (Song et al. 2017). A total of 4312 SNPs were successfully genotyped for 5176 RILs after elimination of 424 RILs including the entire NAM46 family that carried a high rate of nonparental alleles. Of the 4312 SNPs mapped in 5176 RILs, 20 SNPs that were not positioned to the 20 chromosomes of the Wm82.a2.v1 assembly were removed. The low-density array dataset containing 5176 RILs with 4292 SNPs was used as a study panel to be imputed. The 40 NAM parents with 46,245 SNPs generated from SoySNP50K and SoyNAM6K and 527,934 SNPs from sequencing and SoySNP50K assay were used as reference panels. All the SNPs from the study panel nested within the SNPs from reference panels. Furthermore, a dataset of 500 unrelated germplasm accessions genotyped with SoySNP50K BeadChip containing 42,509 SNPs was used to study the quality of imputation in a germplasm population (a population with no direct or unknown genetic relationship) among software (Table S1).

Genotype imputation and QTL mapping may require high-quality genetic map. In previous study, a high-density genetic linkage map was constructed based on 21,478 SNPs for 1083 F_5_-derived RILs genotyped with SoySNP50K BeadChip in the Williams 82 × PI479752 (WP) population (Song et al. 2016). Based on this genetic map, linear interpolation was used to estimate genetic positions of any NAM SNP that was not contained in the WP map according to the SNP physical position according to Diers et al. (2018). The inferred high-resolution genetic maps were subsequently used for SNP imputation and QTL linkage mapping.

### Imputation pipeline

We compared imputation results of population-based imputation vs. bi-parental-based imputation tools under different population structures. For the population-based imputation, the RILs in all 39 NAM families were jointly imputed with the 40 parents as a reference population. For the bi-parental-based imputation, the RILs in each NAM family were imputed with their parents as references based on pedigree information. Because BEAGLE 5.0 and IMPUTE 5 do not take into account pedigree information, they were performed for population-based imputation. While AlphaPlantImpute considers features of bi-parental population and especially the information of parent-progeny relationship, it was used for bi-parental-based imputation. The imputation accuracy of software was compared based on different numbers of markers in study panels, which were 5, 10, 20, 50, 80, 120 and 160 SNPs randomly selected from each of 20 chromosomes across all the RILs. A total of 10 permutations were performed for each marker number. The dataset containing 46,245 SNPs for the 40 parents genotyped with the SoySNP50K and SoyNAM6K Beadchips was mainly used as the reference panel to study effects of factors on imputation accuracy.

Because of the limited number of recombination events within any given biparental population, the majority of haplotype clusters from parents were preserved in progeny for the NAM population, which was beneficial for genotype imputation. Due to the differences of population structure, the imputation performance based on related individuals could be very different from that based on unrelated individuals. Thus, we compared imputation performance of the software for unrelated individuals by simulating a dataset with ungenotyped markers and datasets without ungenotyped markers but with random sporadic missing data points for a germplasm population. To simulate a dataset with ungenotyped markers, we randomly selected 100 from the 500 soybean accessions and randomly masked 10% and 50% SNPs for all the 100 accessions, in addition, we generated reference populations with sizes of 25, 50, 100, 200, 400 from the remaining accessions. The simulation was repeated 10 times for each sample size. For datasets without ungenotyped markers, we used all the 500 accessions but randomly masked 10%, 20%, 30%, 40% and 50% SNPs at sporadic points, which corresponded to imputation without adding markers. The masked genotypes were treated as true genotypes and used to compare with imputed genotypes. The accuracy of imputation at each masked percentage was calculated based on 10 simulations.

### Calculation of imputation accuracy

After randomly selecting a predetermined number of markers in the NAM progeny to simulate different marker density datasets, the remaining markers were treated as true genotypes, which were later used to compare with imputed genotypes. We calculated the correlation coefficient between true and imputed markers after coding the genotypes as 0, 1 and 2 for reference homozygous, heterozygous and alternative homozygous, respectively, per Hickey et al. (2012). Missing genotypes in the true dataset were excluded from the calculation. The average correlation coefficient across SNPs was used to assess the imputation performance for each scenario.

### Optimization of software imputation parameters for soybean populations

**Fig. 1:**
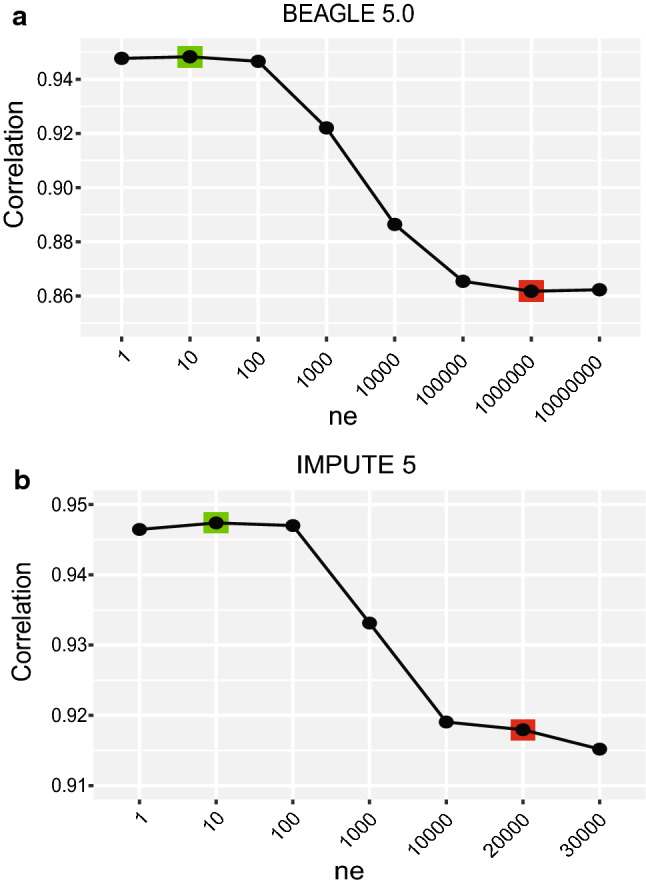
The effect of parameter *ne* on imputation accuracy for BEAGLE 5.0 **a** and IMPUTE 5 **b**. The tests were conducted for the study panel with 50 SNP per chromosome. The red and green squares separately indicate imputation accuracy under default and adapted setting

We tested different BEAGLE 5.0 and IMPUTE 5 parameters that can affect the size of haplotype cluster in the study panel at a marker density of 50 per chromosome. The parameter *ne* (effective population size) in BEAGLE 5.0 can significantly affect the structure of the haplotype cluster, a lower value of *ne* is more suitable for highly related individuals and inbred population (Pook et al. 2020). We tested a number of *ne* specification between 1 and 10,000,000 (1, 10, 100, 1000, 10,000, 100,000, 1,000,000, 10,000,000, default: 1,000,000) and obtained the highest accuracy using *ne* = 10 for soybean, which was 9.50% higher than that using the default *ne* (86.18% accuracy) (Fig. 1a). The parameter *window* specifies the length of sliding window and can be used to control memory consumption. In this study, a larger *window* not only improved imputation accuracy (Fig. S1), but also insured the successful completion of procedure run, especially when imputed samples had extremely low-density markers. We also tested some other parameters with adapted *ne*, such as *err* and parameters that were particularly for ungenotyped markers (*imp-segment, imp-states and cluster*). Except for *err* and *imp-segment*, other parameters did not exist any obvious effect on imputation accuracy compared with the default settings (Fig. S1). Therefore, parameter settings of “*ne* = 10 *window* = 200 *err* = 0.0005 *imp-segment* = 30” in BEAGLE 5.0 were chosen for the imputation.

As a two-step imputation approach, IMPUTE 5 handles pre-phased genotype data which contains no missing alleles. SHAPEIT was used for phasing both the reference and study panels with the parameter setting of “*states* 200 *window 10 effective-size 10*” and the phased results were indexed by Tabix (Li 2011). Similar to BEAGLE 5.0, the parameter *ne* had a marked effect on imputation accuracy. After testing the parameter *ne* from 1 to 30,000 (1, 10, 100, 1000, 10,000, 20,000, 30,000, default: 20,000), we observed a maximum improvement of 4.01% when *ne* was set to 10 compared with the default *ne* (20,000) (91.79% accuracy) (Fig. 1b). In addition, IMPUTE 5 provides two algorithms, divergence selection and neighbor selection, to select the best matching haplotypes to the target haplotype from the reference panel using the Positional Burrows Wheeler Transform (PBWT). We tested these two algorithms by setting *pbwt-depth* values from 4 to 64 under *ne* = 10. Both algorithms achieved the highest imputation accuracy when setting *pbwt-depth* to 64 (default: 8), but the accuracy was slightly higher (0.10%) for the neighbor selection algorithm than the divergence selection (Fig. S2). Based on these results, we chose the parameters “*ne* 10 *pbwt-depth* 64” with the default neighbor selection algorithm to perform imputation in IMPUTE 5.

The parameter *GenotypeSnpThreshold* in AlphaPlantImpute specifies the threshold of minimum proportion of the study individuals genotyped by low-density markers (Gonen et al. 2018). We used a threshold value of 0.9, i.e., only the SNPs that were genotyped in at least 90% of the study individuals were considered as informative markers for imputation. In addition, we set the parameter *PedigreeErrorGenoThreshProp* to 0.05 as the Type 1 error threshold for inferring incorrect assignments of progeny or parents in each bi-parental population by checking Mendelian inconsistencies. AlphaPlantImpute algorithm speculates potential recombination regions and leaves the genotypes around the regions as missing, which can be partially filled using the information of the two closest neighboring markers in the final step by setting the parameter *SnpDistanceFillThreshold* or replaced by the average genotype dosages of the two parent alleles using parameter *FillMissingParentAverage*. We used the genetic map and set the threshold distance of *SnpDistanceFillThreshold* at 0.01 Morgan for filling. Meanwhile, we compared the imputation accuracy without filling vs. with filling using parameter *FillMissingParentAverage.*

### Posterior genotype probability cutoff value

BEAGLE 5.0 and IMPUTE 5 provide the likeliest genotypes in the imputed output, which denote the alleles with the highest posterior genotype probability (GP) at each variant. A GP threshold can be specified to filter imputed genotypes. Previous studies have shown that filtering with GP obviously improved imputation accuracy (Happ et al. 2019; Bolormaa et al. 2019). We explored different GP filtering thresholds from 0.5 to 0.9 to test the effect on imputation quality.

### Linkage disequilibrium extent and minor allele frequency on the imputation accuracy

Previous studies indicated that extensive LD and large haplotype blocks occurred in soybean genome during domestication and artificial selection (Phillips et al. 2003; Song et al. 2015). The LD level reflects the allelic linkage between variants which may affect the imputation accuracy. In order to investigate the LD on imputation accuracy, we calculated the *r*^2^ statistic between imputed and the closest markers in the study panel for population-based imputation using BEAGLE 5.0 and IMPUTE 5, followed by the comparisons of imputation accuracy under different levels of *r*^2^. The *r*^2^ statistic was measured using Plink1.9 (Purcell et al. 2007) between the imputed marker and the closest marker in the study panel identified by genetic distance (He et al. 2015). The imputed markers were divided into ten groups, each group was corresponding to an LD interval from 0.0 to 1.0 with an increment of 0.1. Additionally, the MAF of masked markers was also calculated to evaluate the effect on imputation accuracy. The markers were classified into five successively distinct groups using the following MAF intervals: [0–0.1], [0.1–0.2], [0.2–0.3], [0.3–0.4] and [0.4–0.5] for imputation accuracy evaluations.

### Effect of genetic linkage map distance on imputation accuracy

Previous research has indicated that map-dependent imputation methods showed a higher accuracy than map-independent method; thus high-quality genetic map construction was recommended (He et al. 2015). The genetic map for the region to be imputed was mandatory in IMPUTE 5, but in BEAGLE 5.0, it would be replaced by assumed recombination rate of 1 cM per Mbp automatically if not otherwise specified. For AlphaPlantImpute, a genetic map can be used to fill in the missing genotype in potential recombination region by setting the parameter of maximum genetic distance to identify the closest neighboring markers. The genetic map position and distance of the SNPs were estimated based on the WP linkage map and SNP positions described above.

### Test of original SNP dataset and high-density imputed dataset on QTL mapping

To evaluate the utility of the imputed dataset to narrow the QTL regions, we performed a linkage mapping analysis using the joint inclusive composite interval mapping (JICIM) method in the ICIMapping 4.2 software (Li et al. 2011). The original dataset and imputed dataset were filtered with MAF < 0.1 and missing > 0.20 in each NAM family before each was further used for QTL mapping. Because the ICIMapping 4.2 software can only handle a limited number of markers (20,000–40,000 SNPs depending on the number of lines), the original dataset with 4312 SNPs and imputed dataset with 29,416 SNPs from NAM parents genotyped with SoySNP50K were further used for joint linkage QTL mapping. The genotypic data were recorded as 0, 1, 2, −1, for non-IA3023 parental homozygous, heterozygous, common parental homozygous alleles and missing genotypes, respectively. Genetic linkage maps were inferred from WP linkage maps. For the purpose of examining the effect of imputed datasets on QTL mapping, we analyzed the dataset containing protein content of the NAM population deposited at the Soybase (https://www.soybase.org/SoyNAM/index.php). According to the description at the Soybase site, the field trials with 140 RILs for each of the 40 populations were conducted in Nebraska, Iowa, Illinois and Indiana. The average of seed protein content across the four environments was calculated as phenotypic data. The JICIM method in ICIMapping 4.2 software was performed for QTL analysis across all the 39 NAM families (Li et al. 2011). The statistical method first used generalized linear models by treating the population and population-by-marker interactions as fixed effects to select markers through stepwise regression, then one-dimension scanning for interval mapping was conducted with adjusted phenotypic values (Li et al. 2011). A scanning step of 1 cM and a probability of 0.001 was used for stepwise regression in the first step. The logarithm of odds (LOD) value was determined by 1000 permutation tests with type I error *α* = 0.05 and the final thresholds of 17.90 and 18.42 were obtained for original dataset and imputed dataset, respectively. An approximately 95% confidence interval of each QTL was calculated by one-LOD drop from the estimated QTL position.

## Results

### Imputation accuracy in different scenarios

**Fig. 2:**
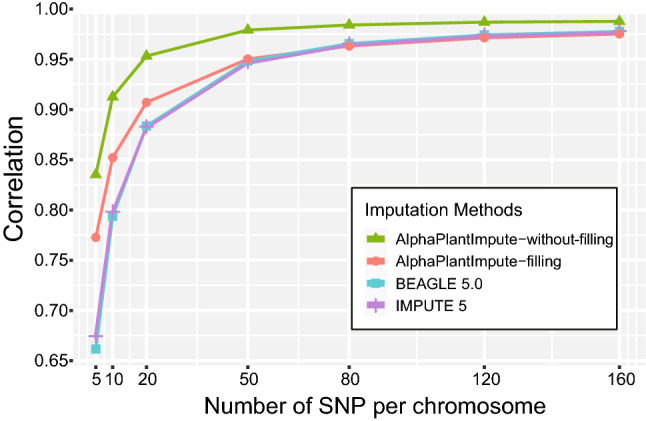
Genotype imputation accuracy based on different numbers of SNPs in study panels. BEAGLE 5.0 and IMPUTE 5 were performed for population-based imputation, while AlphaPlantImpute was performed for bi-parental-based imputation. The imputation accuracy of filling and without-filling in AlphaPlantImpute were displayed

The number of markers in the study panel is an important factor affecting imputation accuracy. As shown in Fig. 2, increasing the number of markers per chromosome from 5 to 160 across all families significantly improved the imputation accuracy of the three software and the effect was more pronounced in the range with low number of markers. The accuracy difference was larger among the imputing methods when the marker density was lower in the study panel. The accuracy plateau reached in all scenarios when imputing with 50 markers per chromosome in the study panels. The average imputation accuracy of the three software increased by 22.03% from 5 to 50 markers, whereas only a slight increase of 2.33% from 50 to 160 markers per chromosome.

BEAGLE 5.0 and IMPUTE 5 showed indistinguishable performances for population-based imputation of the NAM RIL population (Fig. 2). For imputation of 5 markers per chromosome in the study panel, the accuracy was 66.15% for BEAGLE 5.0 and 67.45% for IMPUTE 5, but increased to 97.76% and 97.79% at 160 markers per chromosome, respectively. We also tried bi-parental-based imputation with AlphaPlantImpute. The algorithm leaves the genotypes as missing in potential recombination region (Gonen et al. 2018), but these missing genotypes can be filled by using parameter *FillMissingParentAverage.* For the study panel with 160 markers per chromosome, the highest imputation accuracy was 98.76% but the resulting dataset had a missing data rate of 3.11% without filling, while a slightly lower imputation accuracy of 97.51% without missing genotypes was obtained with filling (Fig. 2).

In general, the AlphaPlantImpute for bi-parental-based imputation without filling had the highest imputation accuracy across all levels of marker density in the study panel (Fig. 2), meanwhile AlphaPlantImpute filling showed a much higher accuracy for the study panel with less than 50 markers per chromosome compared with population-based imputation.

### Effect of filtering with posterior genotype probability on imputation accuracy

**Fig. 3:**
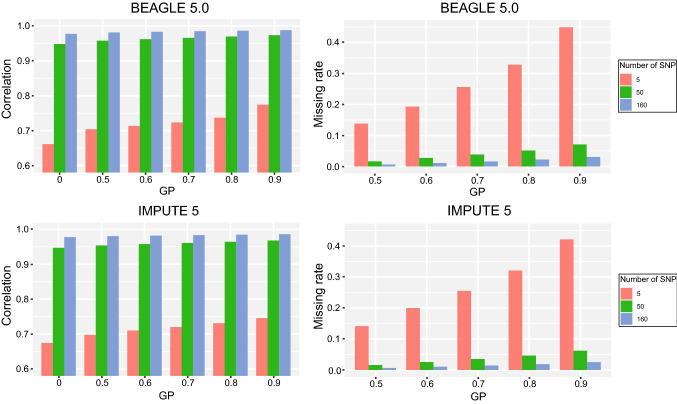
The imputation accuracy and missing rate under different genotype probability cutoff values from 0.5 to 0.9 for BEAGLE 5.0 and IMPUTE 5. GP = 0 means without GP filtering. The tests were conducted for study panels with 5, 50 and 160 SNPs per chromosome

Posterior GP of each imputed genotype was provided by BEAGLE 5.0 and IMPUTE 5. We tested GP cutoff values from 0.5 to 0.9 on imputation accuracy in BEAGLE 5.0 and IMPUTE 5 for NAM RILs. As the GP cutoff value increased, the imputation accuracy increased in varying degrees, but more missing points were reintroduced (Fig. 3). Overall, the increase in accuracy was more significant for imputing with less markers in a study panel. For imputation of 5 and 160 markers per chromosome in study panels performed by BEAGLE 5.0, the accuracy increased by 11.30% and 1.04% when filtered with GP > 0.9 versus without GP filtering. Similar results were obtained for IMPUTE 5 under the same conditions (Fig. 3). Filtering the imputed dataset with GP > 0.9 achieved the highest imputation accuracy of 98.16% and 98.08% but introduced 3.06% and 2.50% missing data in BEAGLE 5.0 and IMPUTE 5, respectively, while the highest imputation accuracy for AlphaPlantImpute was 98.76% with 3.11% missing data rate. Compared with the population-based imputation filtered with GP > 0.9, bi-parental-based imputation still showed slightly better performance.

### Effect of linkage disequilibrium, minor allele frequency and genetic map distance information on imputation accuracy

**Fig. 4:**
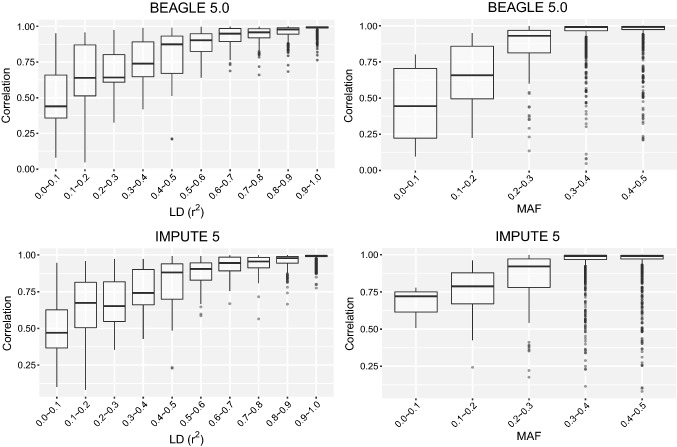
The effect of linkage disequilibrium and minor allele frequency for BEAGLE 5.0 and IMPUTE 5. The tests were conducted for the study panel with 50 SNPs per chromosome. The linkage disequilibrium was measured by *r*^2^ between imputed marker and the closest marker in the study panel. The minor allele frequency was calculated for imputed markers

For both BEAGLE 5.0 and IMPUTE 5 methods, the imputation accuracy in each group of NAM RIL population significantly increased with increasing levels of LD and MAF, though the accuracy difference between the imputing methods was more significant when LD and MAF were low (Fig. 4). When LD level was from [0.0, 0.1] to [0.9, 1.0], the average accuracy of imputation increased from 47.94 to 98.80% and from 49.96 to 98.74% in BEAGLE 5.0 and IMPUTE 5, respectively. When MAF was lower than 0.1, the average accuracy of imputation was 45.37% for BEAGLE 5.0 and 66.96% for IMPUTE 5; however, when MAF was greater than 0.4, the accuracy significantly increased to 95.97% and 95.60% for BEAGLE 5.0 and IMPUTE 5, respectively. The imputation accuracy was generally low for the SNPs with MAF less than 0.2. Imputation accuracy was slightly decreased for BEAGLE 5.0 without using genetic map distance, and the influence varied from 0.03 to 0.86% depending on the marker density of the study panel (Fig. S3).

### Imputation performance of software for a germplasm population

**Fig. 5:**
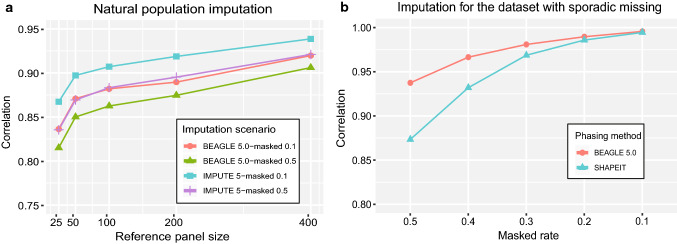
Imputation performance for a germplasm population. **a** The 100 accessions from 500 soybean accessions were randomly masked 10% or 50% SNPs for all individual entries to simulate study panels. Different numbers of individuals were used to study the effect of reference panel size. **b** Genotype data of different missing levels from 50 to 10% were simulated to study the imputation performance for datasets with sporadic missing

We also tested imputation performance for unrelated individuals in a germplasm population based on 500 soybean accessions genotyped with 42,509 SNPs. Two types of datasets were simulated: one with un-genotyped markers and another with completed markers but random sporadic missing genotypes. A higher imputation accuracy was obtained with higher density markers in a study panel (Fig. 5a). The average accuracy increased by 1.29% for BEAGLE 5.0 and 1.69% for IMPUTE 5 when imputation was performed with 10% versus 50% masking markers in study panels. Increasing the reference population size improved the imputation performance. The accuracy improvement of the two imputation methods varied from 6.01 to 8.29% with the reference population size from 25 to 400. The accuracy difference with 10% versus 50% masking markers was more pronounced when reference population size was small. Unlike the indistinguishable performance of BEAGLE 5.0 and IMPUTE 5 for imputing NAM population, the IMPUTE 5 performed slightly better for the germplasm population. For example, the accuracy was up to 95.05% for BEAGLE 5.0 and 96.19% for IMPUTE 5 with 400 individuals in the reference panel and 10% markers masked for the study panel. Sporadic missing genotypes are imputed during phasing. As a phasing tool recommended by the authors of IMPUTE 5 (Rubinacci et al. 2019), SHAPEIT was used to impute the dataset with sporadic missing genotypes and compared with BEAGLE 5.0. Both methods showed significant improvement in imputation accuracy as the missing data rate reduced (Fig. 5b). Accuracy difference was 6.40% between the two methods at 50% missing data rate but gradually narrowed to 0.12% at 10% missing data rate. The accuracy was up to 99.56% for BEAGLE 5.0 and 99.44% for SHAPEIT under 10% masking genotypes.

### Imputed dataset for public access

Based on the AlphaPlantImpute, we imputed the genotypes of the 5176 NAM RILs using the 40 NAM parental genotypes from GBS and SoySNP50K BeadChip assay. A dataset consisting of 5176 RILs with 423,419 SNPs was created. In addition, the linkage map position of each imputed SNP was inferred. The information is deposited at the Soybase (https://soybase.org/SoyNAM/index.php).

### The effect of imputation on linkage analysis

**Fig. 6:**
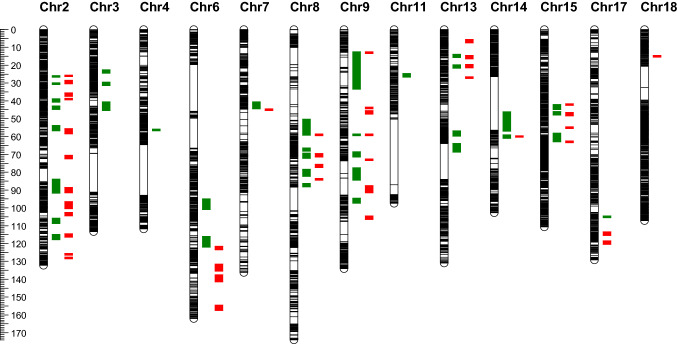
QTL identified for soybean seed protein content in joint linkage analysis. The QTLs detected from original dataset and imputed dataset based on SoySNP50K BeadChip were marked on chromosomes by the confidence intervals. Green square for original dataset and red square for imputed dataset

The original dataset and imputed dataset from AlphaPlantImpute based on NAM parents genotyped with SoySNP50K were further used for joint linkage QTL mapping. The phenotypic variation for soybean seed protein content evaluated at four locations showed normal distribution in all environments. The joint linkage mapping results from the two datasets were merged in Table S2. A total of 36 QTLs were detected on 12 of the 20 chromosomes with phenotypic variation explained (PVE%) of protein content ranging from 3.13 to 23.94% when mapping with original genotype dataset. In imputed dataset, JICIM identified 40 QTLs on 10 of 20 chromosomes with PVE% from 3.96 to 9.86%. More QTLs were discovered on chromosomes 2, 6, 8, 9, 13 and 15 than other chromosomes. The QTLs identified from original or imputed datasets were marked on chromosomes by the confidence intervals using MapChart (Voorrips 2002) (Fig. 6). The average confidence interval of QTLs was 3.94 cM using original dataset and 1.85 cM for imputed dataset. QTL mapping with imputed SNPs significantly narrowed the QTL intervals.

## Discussion

In this study, three software programs were used to impute marker genotypes in 5176 RIL progeny based on high-density marker genotyping information collected on their 40 parents in a soybean NAM population. AlphaPlantImpute is more suited for use in bi-parental population as it can track inheritance of parental alleles and haplotype to progeny. More importantly, AlphaPlantImpute is specifically designed for imputing genotype markers from low-density to high-density, while other pedigree-based imputation methods, such as Tassel-FSFHap, LB-impute, and NOISYmputer, are designed only for imputing sporadic missing points in the dataset, but not for adding more markers to the dataset based on parent or other genotypes. BEAGLE 5.0 and IMPUTE 5 rely on linkage disequilibrium to determine the best matching haplotype between study and reference panels, and are particularly useful with larger size reference panels. Based on the genetic structure of the NAM population, we simulated two scenarios according to the characteristics of the three algorithms: bi-parental-based imputation in every single NAM family using AlphaPlantImpute versus a population-based imputation of the entire NAM population using BEAGLE 5.0 and IMPUTE 5. As expected, imputation with the pedigree information by AlphaPlantImpute had a higher accuracy than population-based imputation by BEAGLE 5.0 and IMPUTE 5, especially when the study individuals were genotyped with extremely low-density markers. In the imputation process, AlphaPlantImpute leaves some genotypes as missing when the algorithm concludes that there may be a potential recombination in the region. Although the missing genotypes could be partially inferred using the information of the two closest neighboring markers or be replaced by the average genotype dosages of the two parents at the expense of reduced imputation accuracy, the accuracy of the non-missing dataset imputed by AlphaPlantImpute was still similar to that of the BEAGLE 5.0 and IMPUTE 5 when the study panel had more than 50 markers per chromosome. In a scenario allowing a small proportion of missing genotypes, imputing the RILs in single NAM family using AlphaPlantImpute with a low value of parameter *SnpDistanceFillThreshold* is suggested.

BEAGLE 5.0 provides two measurements Dosage R-Squared (DR2) and GP to estimate the imputation quality, both of which have been proven to be effective in filtering unreliable imputed genotypes (Nyine et al. 2019; Pook et al. 2020). We choose GP as screening criteria because it discards low-quality sporadic data points rather than particular markers flagged by DR2. Increasing the GP cutoff value could improve the imputation accuracy, but has the tradeoff of reintroducing missing genotypes. This trend was more pronounced when imputed the study panel with extremely low-density markers. The threshold of GP > 0.8 was applied in genotype imputation of BEAGLE4.0 for the wheat GBS dataset and resulted in an accuracy greater than 98.00% and missing data rate lower than 30% (Nyine et al. 2019). Happ et al. (2019) filtered the soybean dataset imputed by BEAGLE4.1 at GP > 0.45 for sequencing depth from 0.1 to 1X and obtained an average of 2.50% accuracy increase at 1X coverage, the data loss was below 5% at all coverage levels. The accuracy improved by an average of 4.26% at GP > 0.9, compared to that at GP > 0.45, but the highest missing data rate up to 20.82% at 0.1X depth. To balance imputation accuracy and reintroduction of missing genotypes, we recommend using the GP cutoff of 0.9 for most datasets.

BEAGLE and IMPUTE were originally designed for use with human populations. In order to improve the compatibility of the software for different species, developers provided many adjustable parameters. Parameter *ne* had a major effect on imputation accuracy in both software. After testing a wide range of *ne* in BEAGLE 5.0 and IMPUTE 5, the highest imputation accuracy was obtained at *ne* = 10 and the accuracy improved 9.50% in BEAGLE 5.0 and 4.01% in IMPUTE 5 at the optimized *ne* compared to the default *ne*. Pook et al. (2020) imputed low-density array SNPs from 50 to 500 K in maize by using BEAGLE 5.0 and obtained the highest imputation accuracy when *ne* = 1000. Because smaller *ne* applies to longer haplotype clusters and highly related individuals, small *ne* is more suitable for imputation in a NAM population. The parameter *window* specifies the width of sliding marker windows (Browning et al. 2018). A large *window* of 200 in this study (default 40 markers) can ensure the program runs smoothly while improving imputation accuracy, especially for the study panel with extremely low-density markers. Several other parameters in BEAGLE 5.0, such as *imp-segment, imp-states and cluster* were also tested with a best-adapted *ne* setting, but had little effect on imputation accuracy although these parameters may affect the structure of haplotype clusters. IMPUTE 5 improves computational efficiency by using PBWT data structure to select reference haplotypes and providing two selection options (Rubinacci et al. 2019). We tested both selection algorithms using different values of *pbwt-depth*. Increasing the value of *pbwt-depth* results in slower runs but a possible increase in accuracy. The highest imputation accuracy was obtained at *pbwt-depth* = 64 (default 8) in both selection algorithms, and accuracy is slightly higher for neighbor selection than divergence selection.

A previous study showed that sequencing depth could be reduced to 0.3X when imputing low-coverage GBS data with BEAGLE 4.1 in soybean while still retaining a high accuracy of 97.80% (Happ et al. 2019). For imputation of non-genotyped markers, the accuracy largely depends on the number of markers on low-density array data that nested to high-density genotyped reference data. In this study, we attempted to find the minimum number of markers on a low-density study panel that did not result in significant loss of imputation accuracy and observed that the imputation accuracy plateau occurred at 50 markers per chromosome in all cases. For AlphaPlantImpute, the imputation accuracy increased from 83.51 to 97.90% by increasing the number of markers from 5 to 50, while only increased from 97.90 to 98.76% by increasing markers from 50 to 160. A similar relationship between imputation accuracy and the number of markers in a study panel was reported by Gonen et al. (2018) in a simulated dataset using AlphaPlantImpute. This result suggested that genotyping the study individuals with 50 randomly distributed markers per chromosome in soybean RIL population was required for high-quality imputation.

The imputation accuracy of population-based methods strongly depends on LD level (Marchini and Howie 2010). Consistent with previous studies (Hickey et al. 2012), imputation accuracy increased with LD level. High imputation accuracy in this study may benefit from the extensive LD and large haplotype blocks in soybean (Song et al. 2015). He et al. (2015) observed a positive correlation between MAF and imputation accuracy, but the effect was far less pronounced than LD. Our results showed a significant positive correlation between MAF and imputation accuracy especially for MAF less than 0.3. In soybean, the euchromatic and heterochromatic regions covered approximately 47% and 53% of the whole genome sequence, respectively (Song et al. 2016). Heterochromatic regions have more extensive LD than euchromatic regions (Song et al. 2015). In this study, of the 4292 SNPs in the NAM RILs, a total of 4042 (94.18%) were from euchromatic regions, whereas 250 (5.82%) from heterochromatic regions. Our study showed that a lower density of markers in the heterochromatic region did not affect the imputation accuracy using the three imputation software programs (Fig. S4). Thus, reducing the number of markers in heterochromatic region when designing the SNP assay will not affect imputation performance but can reduce genotyping cost.

Imputing using genetic map distance is highly recommended in the BEAGLE 5.0 manual and is required in IMPUTE 5. A genetic map in AlphaPlantImpute can be used to fill in the missing genotype in potential recombination regions (Gonen et al. 2018). The imputation accuracy marginally increased with inferred genetic map distance compared to no genetic map distance in BEAGLE 5.0 but the accuracy increase was much larger for the study panel with a lower marker density, which indicated the usefulness of genetic map in low-density genotyping panels. Pook et al. (2020) tested several imputation scenarios using BEAGLE 5.0 with or without genetic map and concluded that imputation with genetic map but without adapted parameters (mainly *ne*) lead to a pronounced reduction in accuracy. To obtain high-quality imputation results, it is suggested to use genetic map and adapted parameters.

Aside from imputation using related individuals, we also studied imputation performance using unrelated individuals in a germplasm population. The imputation accuracy improved significantly when the reference panel size increased from 25 to 50, but then reached plateau. Similar trends were observed for the imputation analysis of maize data by using different versions of BEAGLE (Pook et al. 2020). Unlike the indistinguishable imputation performance for the NAM population, IMPUTE 5 performed better than BEAGLE 5.0 for the germplasm population. Furthermore, imputation accuracy under 10% masking markers was higher than masking 50% markers, and the accuracy difference decreased with increasing reference population size. This is because a larger reference panel may provide more available haplotypes, which significantly increases the possibility of finding the best matching haplotype in the study panel with low-density markers. Even though imputed with large reference panel size and high-density markers in the germplasm population study panel, its highest accuracy was still lower than that achievable in a NAM population, for which the imputation accuracy was higher based on related individuals than unrelated individuals. For dataset with sporadic missing genotypes, BEAGLE 5.0 had a better phasing performance than SHAPEIT especially for the study panel with a high marker missing rate. Imputation for the sporadic missing genotypes often showed a higher accuracy than the imputation for un-genotyped markers.

Soybean seed protein content is a complex quantitative genetic trait controlled by multiple genes and affected by genotype and environment interaction (Chaudhary et al. 2015). Currently, 248 seed protein QTL have been identified to date (February, 2021) in published linkage mapping studies using bi-parental population, according to the Soybean Genetics Committee (http://www.soybase.org). These QTL are distributed on all 20 chromosomes and some of them have been repeatedly detected in different populations (Patil et al. 2017). Although a number of seed protein QTL were detected, many QTL intervals are still large. In our linkage analysis, many QTL were consistently detected using original and imputed datasets. Most importantly, the intervals of most QTL were greatly reduced using the imputed dataset. For example, two intervals of significant QTLs on chromosome 9 were narrowed from 21 to 1 cM and 3 to 1 cM and both of them were overlapped by previously documented QTLs (Hyten et al. 2004; Eskandari et al. 2013). The most significant QTL peak from original dataset was detected at position 59 cM on chromosome 9 and flanked by the two markers at 20.55 and 21.72 Mbp, while the same QTL was positioned in the region of 21.44 to 21.49 Mbp using imputed dataset. This QTL was overlapped with qPR-2 previously reported by Teng et al. (2017). Only two gene models exist in this narrowed region, Glyma.09g109600 is a plant protein of unknown function and Glyma.09g109700 is related to DNA binding transcription factor activity. On chromosome 6, a QTL flanked by two markers located in 18.32 to 46.83 Mbp region using original dataset was mapped in the region from 46.60 to 46.83 Mbp using imputed dataset, and the confidence interval narrowed from 6 to 2 cM. The region was overlapped with QSPC2_1 listed on Soybase (Mao et al. 2013). Most QTLs were detected on chromosome 2, of which 8 from original dataset and 12 from imputed dataset. A QTL interval overlapped with Prot 21–4 (Kabelka et al. 2004) on this chromosome was narrowed from 2 to 1 cM (interval between flanking markers narrowed from 1.21 to 0.05 Mbp) by using imputed dataset. This narrowed interval contained three gene models: Glyma.02g095400 encoding cation efflux protein and zinc transporter, Glyma.02g095500 regulating sulfate transmembrane transporter activity and Glyma.02g095600 encoding histone acetyltransferase. Other two QTL intervals on chromosome 2 overlapped with qPRO8-1 (Qi et al. 2014) and QSPD1b_1 (Mao et al. 2013) were narrowed from 8 to 3 cM (interval between flanking markers narrowed from 9.54 to 0.28 Mbp) and 3 to 2 cM (interval between flanking markers narrowed from 1.16 to 0.36 Mbp), respectively. Major QTLs of soybean seed protein content were frequently detected on chromosome 20 and 15 based on previous studies (Patil et al. 2017). In this study, 3 and 4 QTLs were detected on chromosome 15 using original and imputed dataset, respectively. A QTL interval on chromosome 15 was narrowed from 3 to 1 cM by using imputed dataset, and the interval was approximate to the chromosome 15 QTL region (6.67–9.59 Mbp) frequently reported (Brummer et al. 1997; Fasoula et al. 2004; Warrington et al. 2015). The major QTL on chromosome 20 was mainly reported from the populations derived from wild and cultivated soybean crosses, in this cultivated soybean derived NAM population, genome sequencing of IA3023 and other parents indicated that these genotypes did not have polymorphisms in this QTL region, which prevented us from detecting the QTL. Joint linkage analysis with imputed dataset significantly increased the mapping resolution and facilitated fine mapping of QTL associated with seed protein content in soybean.

The soybean NAM population has been widely used to dissect complex traits (Diers et al. 2018; Scott et al. 2019; Buezo et al. 2019). The imputed high-density dataset for the NAM population and the inferred SNP linkage map position deposited at the public domain are anticipated to be a useful source for the soybean community to perform downstream or other QTL studies.

## Conclusions

Imputation in each NAM family using AlphaPlantImpute had a higher accuracy than imputing all families jointly using either BEAGLE 5.0 or IMPUTE 5. An imputing study panel with at least 50 markers per soybean chromosome is required for suitable soybean population imputation. LD and MAF significantly affect the accuracy of imputation, MAF of SNPs lower than 0.2 generally led to low imputation accuracy. Due to the extent LD in the heterochromatic regions, a very small number of markers are required to obtain high-quality imputation results. A dataset containing > 426,000 SNPs across 5176 RILs and 40 parents was created by imputing the NAM RIL genotypes with AlphaPlantImpute, and is available for public access. Linkage mapping of the QTL controlling protein content showed that using the imputed dataset could narrow the QTL intervals. The study provides useful information for accurate genotype imputation in soybean population and high-quality dataset for identification of QTL controlling other complex quantitative traits.

## Supplementary Information

Below is the link to the electronic supplementary material.

Supplementary file1 (PDF 543 KB)

Supplementary file2 (PDF 274 KB)

Supplementary file3 (XLSX 16 KB)

## Data Availability

Imputed datasets containing 423,419 markers and 29,416 markers for 5176 lines and 40 parents was deposited and available at the Soybase (https://soybase.org/SoyNAM/index.php).
