# Supplementary material for: Genotype imputation for soybean nested association mapping population to improve precision of QTL detection
Source: Theor Appl Genet. 2022 Mar 11;135(5):1797–810. doi: 10.1007/s00122-022-04070-7 (PMC9110473; doi:10.1007/s00122-022-04070-7)
Supplement: Supplementary file 1 — Supplementary file1 (PDF 543 KB) [file 122_2022_4070_MOESM1_ESM.pdf]

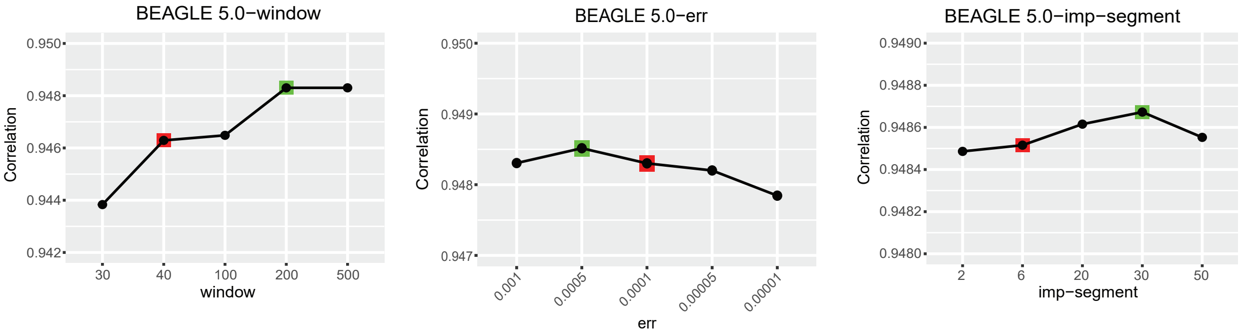

Fig. S1 The effect of parameters window, err and imp-segment on imputation accuracy for BEAGLE 5.0. The tests were conducted for the study panel with 50 SNP per chromosome. The red and green squares separately indicate imputation accuracy under default and adapted setting

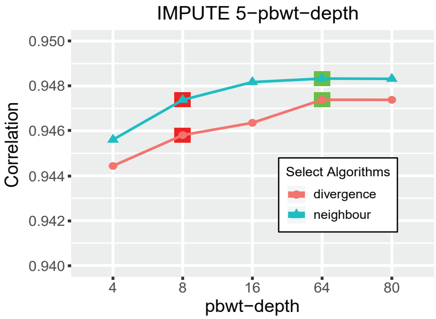

Fig. S2 The effect of parameter pbwt-depth on imputation accuracy based on two selection algorithms for IMPUTE 5. The tests were conducted for the study panel with 50 SNP per chromosome. The red and green squares separately indicate imputation accuracy under default and adapted setting

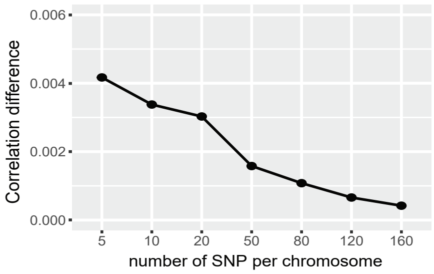

Fig. S3 The difference of imputation accuracy performed by BEAGLE 5.0 with or without genetic map. The accuracy of imputation with genetic map was marginally higher than that without genetic map

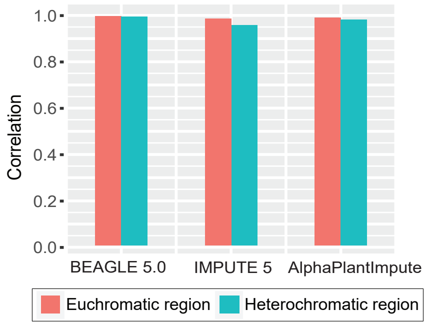

Fig. S4 The imputation accuracy in euchromatic and heterochromatic regions. The total numbers of markers on the study panel were 3003 for euchromatic region and 197 for heterochromatic region
