# Supplementary material for: Genotype imputation for soybean nested association mapping population to improve precision of QTL detection
Source: Theor Appl Genet. 2022 Mar 11;135(5):1797–810. doi: 10.1007/s00122-022-04070-7 (PMC9110473; doi:10.1007/s00122-022-04070-7)
Supplement: Supplementary file 2 — Supplementary file2 (PDF 274 KB) [file 122_2022_4070_MOESM2_ESM.pdf]

Table S1 The origin, name and maturity group of a set of 500 accessions used to study the imputation accuracy

PI	Country of Origin	Cultivar	Maturity Group
PI253651A	China	No. 2	IV
PI587583B	China	ang pu huang da dou	VI
PI200462	Japan	Daizu No. 1	VII
PI407999_1	South Korea	KAERI 544-5	V
PI437615A	China	DV-2788	Z
PI507396	Japan	Tousan 58	IV
PI594758C	China	Xiao ke qing	VIII
PI594764	China	Bai se xuan 3-2	
PI605800A	Vietnam		IV
PI605846B	Vietnam		IV
PI612734	China	Jihei 45	I
PI615446	Vietnam	Hoang mai	IV
PI647086	United States	N8001	VIII
PI036653	China		I
PI063271	China	1187	I
PI065354	China	otanical Garden no. 18	II
PI087059	South Korea	Moyashimame	IV
PI157416	South Korea	Chung-buc-tae	III
PI184042	Yugoslavia	Nikogri	I
PI408055B	South Korea	KAERI 576-3	IV
PI437459	Russia	Ussurijscaja 660	III
PI437988	China	VIR 1624	I
PI438099	China	VIR 2569	I
PI438245	China	VIR 4589	I
PI467323A	China	Jiu nong 13	Z
PI475822A	China		I
PI476887	Vietnam	Cuc Tuyen	III
PI507672	Russia	Avrora	ZZ
PI507689	Russia	Lumina	ZZ
PI507679A	Russia	Harkovcanka	ZZ
PI518754	Yugoslavia	NS-L-118	I
PI549066	Japan	Kitanosuzu	I
PI603387	China	Da jin huang	II
PI603547	China	Xiao hei dou	IV
PI605845A	Vietnam		IV
PI605846F	Vietnam	Sample 115	IV
PI381685	Uganda	X B1	VI

PI423822	South Korea	KAS 360-10	VI
PI441378	Indonesia	Sri Nyonyah	VIII
PI458192	South Korea	KAS 544-29	V
PI567233	China	W6 4507 B	V
PI567316B	China	Hong huang dou	VI
PI587854B	China	Duan jia ai jiao huang	VIII
PI587905	China	Xiao huang dou	VII
PI587915C	China	Bai mao jian	VIII
PI587930	China	Qiu dou	VIII
PI587941	China	Chang ting xi dou	VIII
PI603563C	China	Hei qi huang dou	V
PI605787B	Vietnam	Sample 50	VIII
PI628892	Brazil	Sertaneja	VI
PI628914	Brazil	BA BR-31	VII
PI639566A	Philippines		VIII
PI307838D	India		IX
PI307866	India	No. 31	X
PI594546	China	Long jiang hei dou	IX
PI548471	unknown	Mamredo	VI
PI547702	United States	L67-2324	IV
PI548205	United States	T221	IV
PI599811	United States	C1943	III
PI644050	United States	G95-Cook2734	VIII
PI647082	United States	G04-Ben229IR-M	
PI092686	China	7881	III
PI070516	China	8298	II
PI086115	Japan	Shihyoekuroheso	II
PI089059	China	6063	II
PI054618	China	No. 58	III
PI464883	China	Jiu Nong 9	I
PI438035	China	VIR 2174	Z
PI092595	China	7790	II
PI088300	China	5689	IV
PI437845B	China	DV-910	II
PI438169	China	VIR 4421	I
PI438348A	Bulgaria	Bolgarscaja 397	Z
PI424154B	South Korea	KAS 643-2	IV
PI437578	China	Chuan-da-dou	III
PI068696	China	113	II
PI438080	China	VIR 2489	II
PI081031_2	Japan	Banseiosayada Mame	III

PI253658C	China	No. 9	I
PI084578	South Korea	S-1	III
PI467330	China	Qin-dou No. 2	II
PI297511	China	Dun nun	I
PI424322	South Korea	KAS 333-8	IV
PI061940	China	503	III
PI561298	China	Bai hua cuo zi	II
PI437374	Russia	Ussurijscaja 267	II
PI467337	China	Tong-nong No. 5	II
PI438233C	China	VIR 4524	I
PI467328	China	Ping-ding-xiang	I
PI483084	South Korea	Suweon 97	IV
PI437883	China	Elita 709	II
PI603375	China	an guo jian ye he jia d	I
PI424255A	South Korea	KAS 202-3	IV
PI423787	South Korea	KAS 236-4	IV
PI424273A	South Korea	KAS 235-12	IV
PI437822	China	Charbin 343/C	I
PI592921	China	Hei nong 37	I
PI430594	China	Chin lung No. 5	Z
PI180502	Germany	Strain No. 23	ZZ
PI361107	Russia	Saliut	ZZ
PI424504A	South Korea	KAS 606-6	IV
PI438225	China	VIR 4516	I
PI092689	China	7884	IV
PI445814	Germany	Kirches Stamm 2026	II
PI592913	Russia	#25 (YY)	II
PI398871	South Korea	KLS 109-2	IV
PI438039	China	VIR 2265	Z
PI567159B	China	He long da dou	II
PI578378	China	Bai pi dou	II
PI437340C	Russia	DVIZ 1477	II
PI082263_2	South Korea	Pepute No. 2	II
PI593956C	China	NEAC 190	II
PI437246A	Moldova	CSchi 1087	Z
PI154195	Netherlands	No. 50	II
PI603324A	China	Da jin huang	Z
PI084914	Japan	Ngoiju	III
PI297522	Hungary	Izj hua	Z
PI578494B	China	Jin dou No. 1	III
PI258386	Poland	N. 1954	ZZ

PI417526	France	Garola Rouest	III
PI506779	Japan	Inasato zairai II	IV
PI567511	China	Yi wo hou	III
PI084973	Japan	Takiya	III
PI257432	Germany	C 14/58	Z
PI069995	China	6802	III
PI437621A	China	DV-2796	I
PI398940	South Korea	KLS 602-1	IV
PI424412	South Korea	KAS 540-6	IV
PI467320	China	Jilin No. 17	I
PI257428	Germany	Soja-C.-St. 1/58	Z
PI398271	South Korea	KAS 171-2	IV
PI437519	Russia	VIR 5626	I
PI567156A	China	He jiao No. 8	Z
PI593950A	China	Ken 84-4009	ZZ
PI424607	South Korea	KAS 681-20	IV
PI438020	China	VIR 1889	Z
PI427106	China	Kung chiao 6602-3	II
PI189906	France	Halton	ZZ
PI361063	Yugoslavia	F. 55-54	III
PI361068	Yugoslavia	F. 61-339	ZZ
PI458826A	China	He Feng No. 23	Z
PI407979	South Korea	KAS 541-4	IV
PI424386B	South Korea	KAS 503-13	IV
PI398757	South Korea	KAS 340-3	IV
PI424249A	South Korea	KAS 200-42	III
PI438147	China	VIR 2886	I
PI378672	Russia	Nepoloeaustaja	II
PI424326	South Korea	KAS 333-12	IV
PI408307C	South Korea	KAERI 625-8	IV
PI437779	China	VIR 1318	I
PI603478	China	Yong fu qing da dou	IV
PI424249B	South Korea	KAS 200-42	IV
PI070247	China	8112	III
PI467312	China	Cha-mo-shi-dou	II
PI567417A	China	Bai gun dou	I
PI597440B	China	Hei nong 39	I
PI086145	Japan	Monbetsu Nagaba daiz	III
PI603437A	China	Huang dou	ZZ
PI407724	China	Sin peh tao	II
PI437623	China	DV-2800	I

PI408169D	South Korea	KAERI 660-11	IV
PI068728	China	257	II
PI424221A	South Korea	KAS 131-6	IV
PI437550B	Uzbekistan	VIR 4879	II
PI506685	Japan	Genzoku	III
PI297521	Russia	Irszuriszkaja	II
PI437070	Russia	A-0521	Z
PI594823	China	Bai huang dou	IV
PI424558A	South Korea	KAS 636-11	IV
PI567780A	China	Tong shan zheng ji dou	IV
PI227325	Japan	Kingen No. 2	I
PI417079	China	Koushurei hakubi	III
PI597425	China	Heng feng No. 9	Z
PI089002_2	China	5947	III
PI398693	South Korea	KAS 330-1	III
PI417135A	Japan	Mamyo 50-2	IV
PI153264	Belgium	Tokyo Dwarf Black	II
PI416747	Japan	A-B	I
PI442006	South Korea	KAS 160-2	IV
PI424291	South Korea	KAS 300-3	IV
PI416751	Japan	A-BD	I
PI153241	France	Rouest yellow	Z
PI417230	Japan	Ou kei 1	IV
PI417384	Japan	Tochigi	II
PI603567A	China	Da huang dou <1>	III
PI437348	Russia	Primorscaja 71	I
PI458266	South Korea	KAS 578-11	IV
PI597411A	China	Jilin 26	I
PI437561	China	Bej-man' N217	Z
PI437586A	China	Crest'janscij A	III
PI398493	South Korea	KAS 232-8)	II
PI567212B	Russia	K-002	Z
PI407975A	South Korea	KAERI 540-4	IV
PI423884	Japan	Karikei 73	II
PI291310C	China		II
PI399022	South Korea	KLS 806-1	IV
PI408108	South Korea	KAS 633-22	IV
PI437270A	Moldova	Errj 424	ZZ
PI323586B	Portugal	Dobrageana	II
PI507025	Japan	Manshuu midori meak	IV
PI594178	Japan	Himeyutaka	I

PI605822	Vietnam		II
PI417268	Japan	Sanryuuiri	II
PI603678B	China	eng xian xiao huang do	IV
PI417296	Japan	Shimabara	I
PI603718B	China	Xin jin bai mao zi	IV
PI054859	China	No. 177	II
PI398695	South Korea	KAS 330-2	IV
PI347540C	Yugoslavia	F59-244	ZZ
PI398981	South Korea	KLS 709	IV
PI458142	South Korea	KAS 355-17	IV
PI417580	Japan	Tokyo	III
PI458282	South Korea	KAS 580-8	IV
PI567152	Japan	Shounai 1	II
PI567317	China	Hua huang dou	IV
PI437279	Moldova	VIR 4923	Z
PI592973	China	Kaiyu No. 10	II
PI597406	China	502	I
PI089060	China	6070	I
PI437260A	Moldova	Dobruzanca 629	Z
PI194653	Sweden	752-1	ZZ
PI189947	France	Grignon 6	I
PI603676	China	yun hong mao you yi	IV
PI360846	Japan	Shiroge-9	IV
PI594469A	China	Huang ke zi	III
PI398641	South Korea	KAS 390-19	III
PI594288	Japan	Tachi suzunari	IV
PI424226	South Korea	KAS 133-2	IV
PI219787	Japan	Ohwu No. 13	IV
PI567240	China	Similar to: He pi dou	II
PI593940	China	He feng 7791	Z
PI408211B	South Korea	KAS 600-10-2	IV
PI417196B	Japan	Nishiarai	I
PI603453	China	Dang nian chen	IV
PI378659	Bulgaria	Dobrudzanka V 4731	ZZ
PI603570A	China	Huang dou <1>	IV
PI594235	Japan	Nourin 2	IV
PI089769	China	7190	IV
PI437798	China	VIR 5505	IV
PI603456	China	Lu shun dou	III
PI567266C	China	imilar to: Pu dou 800	III
PI506635	Japan	Choutan daizu	IV

PI605826C	Vietnam	Sample 90	IV
PI506590D	Japan	Bansei ao daizu	IV
PI437478	Russia	VIR 389	IV
PI567660A	China	Tong xu xiao zi huang	IV
PI437683	China	Pa-man-tsin	IV
PI567456	China	Hui bei jia huang dou	IV
PI246369	Japan	Ugo	IV
PI567287	China	Bai gun dou	IV
PI603755B	China	Liu yue dou	III
PI445806	Germany	Herb 606	ZZ
PI603496B	China	Xiao hong dou	IV
PI361110	Romania	Secca	ZZZ
PI464877	China	Mo Shi Dou Gong Di N	III
PI603662B	China	Xiang xiang qing dou	II
PI189873	France	Miko Saumon	Z
PI417476	Japan	Yori karami	Z
PI079694	China	N3	I
PI603428D	China	Da li hei dou	III
PI603637B	China	Qing pi cao huang dou	IV
PI567261D	China	Similar to: Gu tian type	II
PI567262E	China	Similar to: Gu tian type	II
PI567264D	China	Similar to: Gu tian type	III
PI437800	China	VIR 5507	IV
PI339734	South Korea		IV
PI594637	China	Huang ke dou No. 8	IV
PI317335	Japan	Koganejiro	I
PI507494	Japan	Tsuru no tomo	IV
PI603474	China	60 Ri huan jia	II
PI378680C	Russia	VNIIMK 9186	Z
PI507424	Japan	Tousan 84	IV
PI437679	China	Nan-cou	IV
PI507570	Japan	Yuuzuru	III
PI437253	Moldova	Dobruzanca 29	Z
PI297500	China	Charbinszkaja 111	I
PI437663	China	Gun'-tszu-lin' 691	II
PI522187	Ukraine	Krasnogradskaja 1	ZZZ
PI196529	Sweden	770-3	ZZZ
PI437309	Russia	Gorscaja 278	II
PI592917	China	Dong nong 42	Z
PI506865	Japan	Karikei 82	IV
PI592974	China	Liao dou No. 10	III

PI567594B	China	Xiao bai jiao	III
PI437448	Russia	Ussurijscaja 628	II
PI532446	China	Bei feng No. 2	ZZ
PI532457	China	An yue dou	III
PI438005	China	VIR 1748	I
PI393538	Japan	Hika anda No.4	III
PI091103	China	6557	IV
PI398750	South Korea	KAS 339-6	IV
PI189878	France	Mandchourie	ZZ
PI578497B	China	Jin yuan	III
PI561336	China	Man di jin	II
PI229359	Japan	Tohoku No. 1	IV
PI507685A	Ukraine	Kirovogradskaja 5	ZZ
PI438264	China	VIR 5035	Z
PI416953	Japan	Ishihara daizu	II
PI417176	Japan	N-BB	IV
PI437100	Russia	DV-0140	Z
PI372416A	Yugoslavia	Kineska	I
PI591503	United States	L90-7978	III
PI547484	United States	L67-1189	IV
PI547468	United States	L65-52	IV
PI547477	United States	L66-183	IV
PI547597	United States	L70-4413	IV
PI540551	United States	Hobbit	III
PI547589	United States	L63-3270	III
PI547644	United States	L78-3263	IV
PI547837	United States	L75-6141	III
PI572294	United States	BARC-11-6-ff	III
PI547613	United States	L74-836	IV
PI583364	United States	Faribault	I
PI548652	United States	Bass	III
PI583295	United States	HC83-193	IV
PI612764	United States	MN0901	Z
PI547695	United States	L65-1274	II
PI509086	South Korea	KAS 380-8	VI
PI603953	United States	Motte	VIII
PI647961	United States	R01-581F	V
PI417581	United States	H-060072	V
PI548370	China	Manchu, Montreal	I
PI547826	United States	L80-5015	II
PI548654	United States	Hill	V

PI644059	United States	G95-Has4243	VII
PI612615	North Korea	Bochon	Z
PI547627	United States	L76-865	IV
PI377578	Taiwan	S.J. 3	VII
PI095860	South Korea	155	VI
PI628961	Brazil	UFV-Araguaia	VIII
PI553047	United States	Gordon	VII
PI629013	United States	S96-2692	V
PI398628	South Korea	KAS 390-16	VI
PI381668	Uganda	Kakira 13	V
PI594425	China	Xiao cao huang dou	VI
PI458150A	South Korea	KAS 363-7	V
PI399042	South Korea	KLS 902-1	V
PI407871	South Korea	KAERI 511-5	V
PI340898B	Thailand	Maejo No. 1	IX
PI587556A	China	iang ning ai jiao huang	VII
PI423755	South Korea	KAS 205-18	VI
PI506835	Japan	Kanbayashi zairai	V
PI158751	South Korea		V
PI634877	United States	L88-5359	Z
PI603696	China	Shu yang qiu dou jia	VI
PI628917	Brazil	BR-37	VI
PI506608	Japan	Chiyo Zairai	VII
PI468374A	India	Manipur	IX
PI594500A	China	Hei dou	VII
PI407953	South Korea	KAERI 503-2	V
PI307850B	India		IX
PI612711B	China	K 93-89	I
PI522236	United States	Thomas	VII
PI200550	Japan	Yashiro Zairai No. 2	VIII
PI399087	South Korea	(1) Cholla Pukdo	VI
PI507380	Japan	Tousan 41	VI
PI537096	United States	Minnatto	Z
PI407879	South Korea	KAERI 511-13	V
PI483251	Brazil	Cristalina	
PI506966	Japan	Kuro Chouhin 15	VI
PI408202	South Korea	KAERI 600-5-1	V
PI587623	China	ing wan da qing dou N	VI
PI578332B	Argentina	OFPEC Rendidora 801	VII
PI416885	Japan	Ginjiro Nagano	VI
PI424510	South Korea	KAS 606-12	V

PI340904B	Thailand	Sansai No. 2	IX
PI587596C	China	i an wu zui dou jia No	VIII
PI417237	Japan	Ouu 7	V
PI507216A	Japan	Seinaiji Zairai	VI
PI548451	unknown	Delsta	VIII
PI507186	Japan	Rikuu 28	V
PI423855	South Korea	KAS 571-9-3	V
PI227214	Japan	Oku-mame	VII
PI238929	Japan	Karihatakiya	V
PI587964	China	Heng shan hong dou	X
PI567067B	Indonesia		IX
PI567033A	Indonesia	MARIF 2606	VIII
PI567083C	Indonesia		IX
PI603189	United States	T339	IV
PI476910	Vietnam	Thai cao	VI
PI507327	Japan	Tamahomare	VI
PI632661A	Vietnam	H 3	IV
PI398302	South Korea	KAS 180-1	V
PI398418	South Korea	KAS 201-7-2	V
PI594767B	China	Zhao ping hei dou	IX
PI506885	Japan	Kinako Mame	VI
PI507085	Japan	Narisuke	VI
PI606394	Vietnam	Eah leo	III
PI416873C	Japan	Fusanari daizu	VIII
PI423911	Japan	Mie Daizu	VII
PI407771	South Korea	KAS 312-3	VI
PI417258	Japan	Rinou	VIII
PI417442	Japan	Usuao	VII
PI593999A	South Korea		V
PI507236	Japan	Shimokusanoshu	VI
PI587686B	China	Xi li huang No. 1	VI
PI408123	South Korea	KAERI 638-4	V
PI171440	China	No. 3093 Green	VI
PI587844B	China	Tong cheng hei se dou	VI
PI567352C	China	Yang yan qing dou	V
PI424394	South Korea	KAS 521-17	V
PI567057	Indonesia		IX
PI417332	Japan	Shirosaya 8	V
PI507035	Japan	Menka Daizu	VIII
PI200488	Japan	Kiro Aki Daizu	VIII
PI284814	Sudan	C.P.I. 15433	VIII

PI587634A	China	Dan yang hei xiang dou	VII
PI628886	Brazil	RS-6 Guassupi	VII
PI506550A	Japan	o chouhin 16 Shiro Ba	V
PI331795	Vietnam		VIII
PI374201	India		X
PI606365	Vietnam	Bach hoa tao	IV
PI506614	Japan	Chouhin Hitashi 3	VI
PI628876	Brazil	Ocepar-14	VI
PI458258	South Korea	KAS 578-3	V
PI208204	Colombia	Java	VIII
PI587574B	China	Wu jin bai hua dou	VI
PI615504	Vietnam	Nguu mao hong	VI
PI175178	Nepal	No. 9592-A	VIII
PI603775A	China	Ba yue dou	VIII
PI587601D	China	Ru gao ba yue bai jia	VI
PI506650	Japan	Dai-ichi Hienuki 4-3	VI
PI594001	South Korea	SY 9514018	V
PI594630	China	Xiao huang pi dou	VI
PI200484	Japan	Kawara	VIII
PI408164	South Korea	KAERI 660-6	V
PI417314	Japan	Shiro Daizu	VIII
PI548353	unknown	Jogun [Ames]	III
PI587814A	China	Ba yue dou	V
PI605844C	Vietnam	Sample 113	V
PI594839A	China	Huang dou	VIII
PI594852A	China	Chun duo luo zi se dou	IX
PI578437B	Vietnam	Cao qua dia hoa tim	VIII
PI566995B	Indonesia	MARIF 2544	VIII
PI567106B	Indonesia		IX
PI603625	China	Bai ba yue zha zi	V
PI594700B	China	Qing huang za dou No.	V
PI594585	China	hua chi huang dou bi	VII
PI200516	Japan	Okute	VIII
PI407766	China	38-1	VIII
PI594458A	China	Huang ke zi	VII
PI548552	United States	Kanrich	III
PI594820A	China	Jiu yue huang	VIII
PI561362	Japan	Tosan 93	V
PI594796	China	Xi bai dou	VIII
PI597664	United States	T333H	II
PI424178A	South Korea	KAS 663-2	V

PI603198	United States	T348	IV
PI578442	Vietnam	DH 4 xanh	VII
PI407885	South Korea	KAERI 521-6	V
PI605887B	Vietnam		V
PI567139B	Indonesia		IX
PI538402B	Taiwan	AV 68	VII
PI578324H	Nepal		VII
PI175186	India	No. 10126	VII
PI566998B	Indonesia	MARIF 2547	VIII
PI322691	Mozambique	Jubiltan 109	IX
PI605871B	Vietnam	Vang muong khoung	V
PI548200	United States	T211H	IV
PI438432	Israel	VIR 5823	IX
PI567345	China	Niu mao huang	VI
PI507207	Japan	Saku Zairai 2	VII
PI578312	Nepal	I-98	VII
PI548628	United States	Wayne	III
PI416775	Japan	Aki Daizu	VII
PI578318A	Nepal		VI
PI506640	Japan	Chuusei 11	VI
PI612721B	China	Jilin 33	Z
PI323575	India	H 67-26	VIII
PI605817D	Vietnam	Sample 81	V
PI567118B	Indonesia		IX
PI441377	Indonesia	Kedele Genjah	VIII
PI567325B	China	Huang dou	V
PI548497	unknown	Yelredo	VIII
PI605772	Vietnam	Sample 35	VIII
PI548572	Canada	Harly	I
PI417358	Japan	airyuu Tsurunoko Daiz	VI
PI567089B	Indonesia	MARIF 2687	VIII
PI587585D	China	n jiang qiu dao huang	V
PI423801	South Korea	KAS 302-8	V
PI587815C	China	Hong mao za dou	VII
PI506777	Japan	Ikki	VI
PI594864	China	Yang yan dou	V
PI507031	Japan	Meigetsu	V
PI567056A	Indonesia	MARIF 2649	VIII
PI628900	Brazil	IPB 90-77	VI
PI567235A	China	W6 4508	VIII
PI507497	Japan	Ueda Tairyuu Mejiro 1	VI

PI603522	China	Gao gan qing	VI
PI507546	Japan	Yamato Zairai	VII
PI567022B	Indonesia		IX
PI458272	South Korea	KAS 579-1	V
PI495018	China	San ming 73-11	IX
PI416926A	Japan	Hishiumi zairai	IX
PI594635D	China	Qing huang za dou No.	VI
PI506753	Japan	Hitashi Mame 2	VI
PI423781B	South Korea	KAS 234-2	V
PI416792	Japan	Ao daizu	V
PI588027D	China	Da huang ke	V
PI548409	Japan	Sato	IV
